# Comparison of dexmedetomidine and propofol for sedation in patients with traumatic brain injury

**DOI:** 10.1186/cc13606

**Published:** 2014-03-17

**Authors:** O Tarabrin, S Shcherbakov, D Gavrychenko, G Mazurenko

**Affiliations:** 1 Odessa National Medical University, Odessa, Ukraine

## Introduction

Both propofol and dexmedetomidine decrease systemic blood pressure, heart rate, and cardiac output in a dose-dependent manner. The aim of this study was to compare their safety and efficacy for intravenous sedation during mechanical ventilation.

## Methods

Eighty-four patients with traumatic brain injury (Glasgow scale 7 to 8) entered the study, mean age 44 ± 13.37 years. All patients underwent mechanical lung ventilation. Patients were divided into two groups depending on the type of intravenous sedation. In the first group (*n *= 42), sedation was performed by intravenous propofol infusion at a dose of 4 to 12 mg/kg/hour. In patients of the second group (*n *= 42), sedation was carried out by intravenous infusion of dexmedetomidine at a dose of 0.2 to 1.4 mg/kg/hour. The level of sedation was assessed with bispectral index monitoring, targeting index 70. For comparison of methods we evaluated heart rate, blood pressure, SpO_2 _30, 90 and 180 minutes after the start of sedation. Both groups were matched for sex, age and comorbidity.

## Results

Thirty minutes after the start of sedation in patients of the first group, HR was 83 ± 11.31 beats/minute, blood pressure was 127 ± 12.87/64 ± 8.54 mmHg, SpO_2 _was 97 ± 3.01%. In patients of the second group 30 minutes after the beginning of sedation, HR was 87 ± 10.01 beats/minute, blood pressure was 131 ± 11.67/68 ± 8.19 mmHg, SpO_2 _was 97 ± 2.98%. After 90 minutes in the first group of patients, we observed HR was 81 ± 6.27 beats/minute, blood pressure was 119 ± 11.46/59 ± 4.29 mmHg, SpO_2 _was 98 ± 2.35%. In the second group, HR was 82 ± 7.31 beats/minute, blood pressure was 94 ± 13.62/55 ± 7.81 mmHg, SpO_2 _was 97 ± 2.76%. In the first group 180 minutes after the start of sedation, HR was 86 ± 6.19 beats/minute, blood pressure was 105 ± 10.34/54 ± 4.28 mmHg, SpO_2 _was 98 ± 1.32%. In the second group, HR was 75 ± 6.27 beats/minute, blood pressure was 92 ± 12.54/51 ± 6.91 mmHg, SpO_2 _was 96 ± 2.91%.

## Conclusion

Using dexmedetomidine at a dose of 0.2 to 1.4 mg/kg/hour for intravenous sedation is safe in terms of hemodynamic stability and blood oxygenation for sedation during mechanical lung ventilation in traumatic brain injury patients.

